# Emerging Role of Corynebacterium in Breast Abscesses: Clinical Profiles, Resistance Patterns, and Recommendations

**DOI:** 10.7759/cureus.103156

**Published:** 2026-02-07

**Authors:** Rufina Soomro, Nikhat Fatima, Sana Anwar

**Affiliations:** 1 Department of Breast Surgery, Liaquat National Hospital and Medical College, Karachi, PAK; 2 Department of Microbiology, Liaquat National Hospital and Medical College, Karachi, PAK

**Keywords:** antibiotic resistance, breast abscess, corynebacterium spp, non-lactational abscess, pathogen susceptibility

## Abstract

Background

*Corynebacterium* species are emerging pathogens in breast abscesses, with unique clinical, pathological, and antibiotic resistance patterns that pose diagnostic and therapeutic challenges. This study aimed to evaluate the clinical, pathological, and microbiological characteristics of *Corynebacterium*-associated breast abscesses, with a focus on antibiotic resistance and evidence-based management.

Methodology

This retrospective observational study over eight years analyzed 42 patients with *Corynebacterium*-positive breast abscesses.

Results

Most patients were ≤35 years old (22, 52.4%), and non-lactating (39, 92.9%). Periductal inflammation was found in 39 (92.3%) patients. Ciprofloxacin and clindamycin resistance was high (39, 92.9%), while vancomycin and linezolid showed 100% sensitivity. The most common species isolated was *Corynebacterium striatum *(n = 9) isolated from the 18 strains of *Corynebacterium* species that were subtyped.

Conclusions

This study underscores non-lactational predominance and high antibiotic resistance in *Corynebacterium*-associated abscesses, advocating targeted therapy based on susceptibility testing.

## Introduction

*Corynebacterium* species, historically dismissed as commensals or contaminants, have increasingly been recognized as true pathogens implicated in a range of infections, including breast abscesses [1]. These gram-positive, lipophilic bacilli, especially *Corynebacterium kroppenstedtii*, are now known to cause indolent but persistent infections that often masquerade as other inflammatory or granulomatous conditions [2]. However, the recognition of their pathogenic potential is a relatively recent development in medical microbiology, challenging earlier perceptions of their clinical insignificance.

Breast abscesses are a significant clinical condition caused by a range of bacterial pathogens, with *Staphylococcus aureus* traditionally considered the most common causative organism. However, emerging evidence highlights the role of *Corynebacterium* species as significant yet under-recognized pathogens in this domain. Among the pathogenic species, *Corynebacterium kroppenstedtii* and *Corynebacterium amycolatum* are particularly notable in breast infections [3]. Unlike more common causes such as *Staphylococcus aureus* or *Streptococcus* species, infections caused by *Corynebacterium* often exhibit atypical presentations. These may include slow-growing, indolent infections, recurrent abscess formation, or minimal inflammatory signs despite substantial underlying pathology [4]. Such unique characteristics frequently lead to diagnostic delays or misdiagnoses, especially in non-lactational breast abscesses, which are less well-understood compared to their lactational counterparts [5].

Predisposing factors such as diabetes, immune suppression, and prior antibiotic exposure are often implicated in *Corynebacterium* infections. These factors may compromise host defenses, allowing *Corynebacterium* to proliferate and cause persistent infections [6]. Histopathological findings in *Corynebacterium*-associated infections often reveal periductal inflammation or granulomatous mastitis, which may mimic other conditions such as tuberculosis or fungal infections, further complicating diagnosis [7]. This highlights the importance of thorough microbiological and pathological investigations to establish accurate diagnoses and initiate targeted treatments.

Antibiotic resistance, in general, [8] and patterns of *Corynebacterium* species, in particular, are another significant concern [9,10]. Resistance to commonly prescribed antibiotics such as clindamycin, erythromycin, and fluoroquinolones is increasingly reported, necessitating careful consideration of antibiotic stewardship and the development of effective treatment protocols [11]. Furthermore, the lack of large-scale studies or robust surveillance data, particularly in resource-limited settings, leaves substantial gaps in our understanding of epidemiology, risk factors, and clinical outcomes associated with *Corynebacterium* infections.

This study aims to bridge these knowledge gaps by examining a cohort of patients with *Corynebacterium*-associated breast abscesses over an extended period. By analyzing their clinical, pathological, and microbiological characteristics, as well as antibiotic susceptibility patterns, this research seeks to contribute to a more comprehensive understanding of these infections. Additionally, by comparing findings with international literature, the study aims to inform evidence-based guidelines for the diagnosis and management of *Corynebacterium*-associated breast abscesses, with a particular focus on optimizing antibiotic therapy and addressing emerging resistance.

Study objectives

This study aimed to evaluate patients’ demographic, clinical, and pathological characteristics with *Corynebacterium*-associated breast abscesses. It also aimed to assess the antibiotic prescribing patterns and susceptibility profiles of *Corynebacterium* isolates. Further, it aimed to explore potential associations between demographic factors (age, lactation status), comorbidities, and histopathological findings. Finally, it compared local findings with international data to refine antibiotic selection and optimize clinical management protocols.

## Materials and methods

Study design and data collection

This retrospective observational study was conducted at Liaquat National Hospital, Karachi. Data for the last eight years were collected from January 2017 to June 2024.

Study design and setting

All patients diagnosed with breast abscesses from which *Corynebacterium* species were isolated on pus culture were considered for inclusion. Patients with breast abscesses caused by other organisms were excluded. The study was conducted collaboratively by the Departments of Microbiology and Breast Surgery, who jointly verified all clinical and laboratory details.

Data collection

Patient medical records were reviewed to obtain demographic details (age, lactation status, comorbidities), clinical presentation, histopathological findings, prior antibiotic exposure, and antimicrobial susceptibility profiles. Only cases with culture-confirmed *Corynebacterium* isolates from breast pus aspirates, discharge swabs, or drainage samples were included. Data accuracy was cross-checked by the Microbiology and Breast Surgery teams.

Sample processing and microbiological methods

All samples were processed in the Microbiology Laboratory following the Clinical Microbiology Procedures Handbook guidelines, which is a validated protocol.

Initial evaluation

Gram stains were prepared with appropriate controls. Samples were cultured on blood agar, chocolate agar, and MacConkey agar. Pure or heavy growth of a single organism, the presence of moderate-to-numerous pus cells, and Gram-positive coryneform bacilli were considered significant for reporting *Corynebacterium* infection.

Identification

All isolates were initially reported as *Corynebacterium* species. Species-level identification was subsequently performed using the VITEK 2 ANC Card (Anaerobic and *Corynebacterium* Identification Card).

Antimicrobial susceptibility testing

Susceptibility was tested using Oxoid discs against penicillin 10 U, vancomycin 30 µg, gentamicin 10 µg, ciprofloxacin 5 µg, clindamycin 2 µg, tetracycline 30 µg, and linezolid 30 µg. Interpretation of inhibition zones followed the EUCAST Breakpoint Tables (Version 15.0, 2025) [12].

Statistical analysis

Statistical analysis was performed using SPSS (IBM Corp., Armonk, NY, USA). Continuous variables were summarized as mean ± standard deviation (SD), and categorical variables were presented as frequencies and percentages. Associations between categorical variables (e.g., comorbidities vs. histopathology, lactation status vs. histopathology, age groups vs. histopathology) were assessed using the chi-square test or Fisher’s exact test when expected frequencies were <5. Continuous variables (e.g., age) were compared using an independent-samples t-test or analysis of variance, depending on variable distribution. All tests were two-tailed, and a p-value <0.05 was considered statistically significant.

Ethical considerations

The study was conducted after the approval of the Research Committee (LNH-Breast Surgery-06.2024/65) and the Ethics Committee of Liaquat National Hospital (approval number: 1084-2024-LNH-ERC). Patients’ identity was kept confidential.

## Results

A total of 42 patients with *Corynebacterium* breast abscesses were included. The mean age was 37.6 ± 12.0 years. More than half (22, 52.4%) were ≤35 years, 14 (33.3%) were 36-50 years old, and 6 (14.3%) were >50 years old. Age distribution was not significantly associated with histopathological patterns (chi-square test, p = 0.40). Similarly, lactation status (3 (7.1%) lactating vs. 39 (92.9%) non-lactating) showed no significant relationship with histopathology (chi-square test, p = 0.45) (Table 1).

**Table 1:** Patient demographics and clinical characteristics (n = 42). Values are presented as mean ± SD or n (%). Analysis of variance (ANOVA) was used for continuous variables, and the chi-square test was used for categorical comparisons. NS = not significant. A p-value <0.05 was considered statistically significant.

Variable	N (%)/Mean ± SD	Statistical test	Value	P-value
Mean age (years)	37.59 ± 11.98	ANOVA	F = 1.12	0.29 (NS)
Age distribution		Chi-square	χ² = 1.84, df = 2	0.40 (NS)
≤35 years	22 (52.4)			
36–50 years	14 (33.3)			
>50 years	6 (14.3)			
Lactating	3 (7.1)	Chi-square	χ² = 0.56, df = 1	0.45 (NS)
Non-lactating	39 (92.9)			

Comorbidities were present in a considerable proportion of patients, including diabetes mellitus (7, 16.7%), hypertension (6, 14.3%), malignancy (5, 11.9%), hypothyroidism (2, 4.8%), and skin conditions (2, 4.8%). To explore potential factors associated with disease severity, associations between demographic variables, lactation status, and comorbidities with histopathological patterns were evaluated.

Age distribution and lactation status showed no significant association with pathology (p = 0.40 and p = 0.45, respectively), indicating that these variables were not major contributors to the observed histopathological patterns (Table 1). In contrast, comorbidities demonstrated a significant overall association with histopathology (p = 0.013). Among individual comorbidities, diabetes mellitus was the only one significantly associated with periductal inflammation (p = 0.033). Hypertension (p = 0.07) and malignancy (p = 0.13) did not reach statistical significance (Table 2).

**Table 2:** Association between comorbidities and histopathological findings. Values are presented as mean ± SD or n (%). Analysis of variance (ANOVA) was used for continuous variables, and the chi-square test was used for categorical comparisons. NS = not significant. A p-value <0.05 was considered statistically significant.

Comorbidity	N (%)	Histopathology (periductal vs. other)	Statistical test	Value	P-value
Diabetes	7 (16.7)	6 vs. 1	Chi-square	χ² = 4.52, df = 1	0.033*
Hypertension	6 (14.3)	6 vs. 0	Fisher’s exact	-	0.07
Malignancy	5 (11.9)	4 vs. 1	Chi-square	χ² = 2.21, df = 1	0.13
Hypothyroidism	2 (4.8)	2 vs. 0	Fisher’s exact	-	0.28
Skin conditions	2 (4.8)	2 vs. 0	Fisher’s exact	-	0.28

Species-level identification via the VITEK 2 ANC card was achieved in 18 isolates. *Corynebacterium striatum* was the predominant species (n = 9), followed by *Corynebacterium amycolatum* (n = 6) and *Corynebacterium minutissimum* (n = 3).

Histopathological examination revealed periductal inflammation as the predominant finding (39, 92.3%), while granulomatous inflammation (2, 3.8%) and lobular involvement (2, 3.8%) were infrequent (Table 3).

**Table 3:** Histopathological findings (n = 42). Frequencies are presented as n (%). No statistical comparison is applicable.

Pattern	N (%)
Periductal inflammation	39 (92.3)
Granulomatous inflammation	2 (3.8)
Lobular involvement	2 (3.8)

Prior antibiotic use was reported in 33 (78.6%) cases. The most frequently prescribed empiric agents were clindamycin (29, 69.7%), co-amoxiclav (26, 60.6%), and ciprofloxacin (26, 60.6%). Susceptibility testing, however, revealed universal sensitivity to vancomycin and linezolid (42, 100% each), moderate sensitivity to penicillin (24, 59.5%), and poor sensitivity to clindamycin (3, 7.1%), ciprofloxacin (3, 7.1%), and erythromycin (2, 4.8%) (Table 4).

**Table 4:** Antibiotic susceptibility profiles of Corynebacterium isolates. Percentages reflect the proportion of isolates sensitive to each antibiotic (n = 42). No statistical test was performed.

Antibiotic	Sensitive isolates (%)
Vancomycin	42 (100)
Linezolid	42 (100)
Penicillin	24 (59.5)
Clindamycin	3 (7.1)
Ciprofloxacin	3 (7.1)
Erythromycin	2 (4.8)

## Discussion

This study highlights the emerging role of *Corynebacterium* species as significant pathogens in breast abscesses, with a predominant presentation in non-lactational cases. The mean age of our cohort was 37.6 years, and more than half of the patients were ≤35 years old, reflecting a younger demographic compared with Western cohorts. While age distribution and lactation status did not show significant associations with histopathological findings (p = 0.40 and p = 0.45, respectively), the marked predominance of non-lactational abscesses (92.9%) supports recent reports of a global epidemiological shift toward chronic, non-lactational disease [13-15].

Comorbid conditions, particularly those affecting immune or metabolic function, may contribute to the rising incidence of *Corynebacterium* breast infections. In our cohort, diabetes showed a significant association with periductal inflammation (p = 0.033), suggesting that impaired immune responses and altered tissue microenvironment may predispose to chronic or atypical infections. Although other comorbidities, such as hypertension and malignancy, did not reach statistical significance (p = 0.07 and p = 0.13, respectively), their presence in a notable proportion of patients indicates that systemic health factors may influence susceptibility. These findings support the hypothesis that underlying host factors, rather than organism virulence alone, play an important role in disease development and chronicity, reinforcing the need to consider comorbid states when evaluating and managing *Corynebacterium* breast infections.

A key finding was the significant association between comorbidities and histopathology (p=0.013). Diabetes showed a significant relationship with periductal inflammation (p = 0.033), suggesting that metabolic and immune conditions may predispose patients to more severe or chronic inflammatory patterns [16]. Other comorbidities, such as hypertension and malignancy, did not reach statistical significance (p = 0.07 and p = 0.13, respectively). These results highlight the need for closer attention to comorbid states in the management of *Corynebacterium* breast infections.

Histopathological analysis was dominated by periductal inflammation (92.3%). This aligns with prior reports linking *Corynebacterium kroppenstedtii *to granulomatous and periductal mastitis [17,18]. Large multicenter series have also described similar histopathological patterns in idiopathic granulomatous mastitis without confirmed microbial identification [19], suggesting that some idiopathic cases may represent unrecognized *Corynebacterium* infection.

Antibiotic susceptibility patterns revealed an important mismatch between empirical prescribing and actual resistance profiles. Clindamycin and ciprofloxacin, among the most frequently prescribed empiric agents, demonstrated poor sensitivity (7.1% each), exceeding previously reported global resistance rates of 40-60% [20]. By contrast, vancomycin and linezolid maintained 100% activity, consistent with international in vitro studies and systematic reviews [21,22]. Penicillin showed moderate susceptibility (59.5%), suggesting it may serve as a cost-effective option in resource-limited settings when supported by susceptibility testing. These results reinforce the importance of culture-directed therapy and antibiotic stewardship [23].

Diagnostic limitations also became evident. Only 18 of 42 isolates could be speciated using the VITEK 2 ANC card, with *Corynebacterium striatum* and *Corynebacterium amycolatum* most frequently identified. However, VITEK 2 is unable to reliably detect *Corynebacterium kroppenstedtii*, which requires lipid-supplemented media or advanced diagnostic tools such as matrix-assisted laser desorption/ionization-time of flight (MALDI-TOF) mass spectroscopy (MS) or 16S rRNA sequencing [24]. Recent reviews emphasize that combining advanced diagnostics with antimicrobial stewardship strategies is essential for accurate pathogen identification and tailored treatment [25].

Overall, this study underscores the importance of recognizing *Corynebacterium* species as true pathogens in non-lactational and chronic breast abscesses. Tailoring antibiotic therapy based on susceptibility testing, considering comorbidities such as diabetes, and strengthening diagnostic capabilities are critical steps to improve outcomes and reduce recurrence.

Limitations

This study had some limitations, such as a single-center design and a small sample size, which limited generalizability, a retrospective design, with risk of incomplete data and misclassification bias. We had restricted identification methods, where the lack of molecular testing (e.g., 16S rRNA sequencing) may have underestimated species diversity. There is no follow-up data, so outcomes and recurrence rates could not be assessed longitudinally.

Future recommendations

We suggest prospective larger multicenter studies with standardized data collection, with incorporation of molecular diagnostics (MALDI-TOF, 16S sequencing). Formal antimicrobial stewardship programs would help ensure rational prescription of antibiotics. Long-term outcome studies would be useful in assessing recurrence and therapeutic strategies.

## Conclusions

*Corynebacterium* species are emerging as true pathogens in chronic and non-lactational breast abscesses, particularly among younger women. Diabetes showed a significant association with periductal inflammation, highlighting comorbidities as important risk factors. The mismatch between empiric prescribing and resistance patterns emphasizes the need for culture-directed therapy and antibiotic stewardship. Vancomycin and linezolid remain reliable, while penicillin may be a cost-effective option in resource-limited settings. Given the limitations of automated systems such as VITEK 2, advanced diagnostics such as MALDI-TOF MS or gene sequencing should be incorporated into clinical practice. Larger multicenter studies are warranted to guide standardized management.

## References

[1] Riegel P, Liégeois P, Chenard MP, Mathelin C, Monteil H (2004). Isolations of Corynebacterium kroppenstedtii from a breast abscess. Int J Med Microbiol.

[2] Stary CM, Lee YS, Balfour J (2011). Idiopathic granulomatous mastitis associated with corynebacterium sp. Infection. Hawaii Med J.

[3] Saraiya N, Corpuz M (2019). Corynebacterium kroppenstedtii: a challenging culprit in breast abscesses and granulomatous mastitis. Curr Opin Obstet Gynecol.

[4] Zeng W, Lao S, Jia W (2022). Clinical features and recurrence of Corynebacterium kroppenstedtii infection in patients with mastitis. BMC Womens Health.

[5] Lu C, Marcucci V, Kibbe E, Patel R (2023). Granulomatous mastitis secondary to Corynebacterium requiring surgical intervention: a complicated diagnosis. J Surg Case Rep.

[6] Leal J, Gregson DB, Ross T, Church DL, Laupland KB (2008). Epidemiology of Clostridium species bacteremia in Calgary, Canada, 2000-2006. J Infect.

[7] Sánchez López ME, Sánchez Mozo A, Juez Barrera JM (2022). Recurrent bilateral granulomatous mastitis due to Corynebacterium tuberculostearicum with mammary scrofula: a case report. Rev Senol Patol Mamar.

[8] Urban-Chmiel R, Marek A, Stępień-Pyśniak D, Wieczorek K, Dec M, Nowaczek A, Osek J (2022). Antibiotic resistance in bacteria-a review. Antibiotics (Basel).

[9] Bernard K (2012). The genus corynebacterium and other medically relevant coryneform-like bacteria. J Clin Microbiol.

[10] Otsuka Y, Kawamura Y, Koyama T, Iihara H, Ohkusu K, Ezaki T (2005). Corynebacterium resistens sp. nov., a new multidrug-resistant coryneform bacterium isolated from human infections. J Clin Microbiol.

[11] Asgin N, Otlu B (2020). Antimicrobial resistance and molecular epidemiology of Corynebacterium striatum isolated in a tertiary hospital in Turkey. Pathogens.

[12] European Committee on Antimicrobial Susceptibility Testing (2015). Breakpoint Tables for Interpretation of MICs and Zone Diameters.

[13] Li S, Huang Q, Song P, Han X, Liu Z, Zhou L, Ning P (2023). Clinical characteristics and therapeutic strategy of granulomatous mastitis accompanied by Corynebacterium kroppenstedtii: a retrospective cohort study. BMC Womens Health.

[14] Goh Z, Tan AL, Madhukhumar P, Yong WS (2015). Recurrent Corynebacterium kroppenstedtii breast abscess in a young Asian female. Breast J.

[15] Sanchez Eluchans N, Barberis C, Cittadini R (2021). [Corynebacterium kroppenstedtii breast infections: report of four cases]. Rev Argent Microbiol.

[16] Taylor GB, Paviour SD, Musaad S, Jones WO, Holland DJ (2003). A clinicopathological review of 34 cases of inflammatory breast disease showing an association between corynebacteria infection and granulomatous mastitis. Pathology.

[17] Wong SC, Poon RW, Chen JH (2017). Corynebacterium kroppenstedtii is an emerging cause of mastitis especially in patients with psychiatric illness on antipsychotic medication. Open Forum Infect Dis.

[18] Hacibekiroglu T, Gokoz Dogu G, Ozturk EB, Korkmaz P, Erbay A, Altundag K (2018). Linezolid in the treatment of breast abscess caused by Corynebacterium kroppenstedtii. Infect Dis Rep.

[19] Co M, Cheng VC, Wei J, Wong SC, Chan SM, Shek T, Kwong A (2018). Idiopathic granulomatous mastitis: a 10-year study from a multicentre clinical database. Pathology.

[20] Dobinson HC, Anderson TP, Chambers ST, Doogue MP, Seaward L, Werno AM (2015). Antimicrobial treatment options for granulomatous mastitis caused by Corynebacterium species. J Clin Microbiol.

[21] Bernard K, Pacheco AL (2015). In vitro activity of 22 antimicrobial agents against Corynebacterium and Microbacterium species referred to the Canadian National Microbiology Laboratory. Clin Microbiol Newslett.

[22] Milosavljevic MN, Milosavljevic JZ, Kocovic AG (2021). Antimicrobial treatment of Corynebacterium striatum invasive infections: a systematic review. Rev Inst Med Trop Sao Paulo.

[23] Shrestha J, Shrestha R, Sapkota S (2023). Antimicrobial Stewardship. http://www.ncbi.nlm.nih.gov/books/NBK572068/.

[24] Claeys KC, Johnson MD (2023). Leveraging diagnostic stewardship within antimicrobial stewardship programmes. Drugs Context.

[25] Oliveira A, Oliveira LC, Aburjaile F (2017). Insight of genus Corynebacterium: ascertaining the role of pathogenic and non-pathogenic species. Front Microbiol.

